# Early changes in myocardial repolarization and coronary perfusion after cardiopulmonary bypass surgery for ASD repair in children

**DOI:** 10.1186/1471-2261-13-67

**Published:** 2013-09-10

**Authors:** Elhadi H Aburawi, Abdul-Kader Souid, Petru Liuba, Taoufik Zoubeidi, Erkki Pesonen

**Affiliations:** 1 Pediatric Department, Section of Pediatric Cardiology, Skane University Hospital, Lund University, Lund, Sweden; 2 Pediatric Department, College of Medicine and Health Sciences, United Arab Emirates University, P.O. Box 17666, Al-Ain, Abu Dhabi, UAE; 3 Department of Statistics, College of Business and Economics, United Arab Emirates University, Al-Ain, UAE

**Keywords:** ECG, ASD, Repolarization, Heart surgery

## Abstract

**Background:**

In adults, impaired myocardial repolarization and increased risk of arrhythmia are known consequences of open heart surgery. Little is known, however, about post-operative consequences of cardiopulmonary bypass surgery in children. The aim of this study was to assess ventricular repolarization and coronary perfusion after bypass surgery for atrial septal defect (ASD) repair in children.

**Methods:**

Twelve patients with ASD were assessed one day before and 5–6 days after ASD repair. Myocardial repolarization (corrected QT interval, QTc, QT dispersion, QTd, and PQ interval) was determined on 12-lead electrocardiograms. Coronary flow in proximal left anterior descending artery (peak flow velocity in diastole, PFVd) was assessed by transthoracic Doppler echocardiography.

**Results:**

Ten of the 12 (83%) children had normal myocardial repolarization before and after surgery. After surgery, QTc increased 1-9% in 5 (42%) patients, decreased 2-11% in 5 (42%) patients and did not change in 2 (16%) patients. Post-op QTc positively correlated with bypass time (*R*=0.686, *p*=0.014) and changes in PFVd (*R*=0.741, *p=*0.006). After surgery, QTd increased 33-67% in 4 (33%) patients, decreased 25-50% in 6 patients (50%) and did not change in 2 (16%) patients. After surgery, PQ interval increased 5-30% in 4 (33%) patients, decreased 4-29% in 6 (50%) patients and did not change in 1 (8%) patient. Post-op PQ positively correlated with bypass time (*R*=0.636, *p*=0.027). As previously reported, PFVd significantly increased after surgery (*p*<0.001).

**Conclusions:**

Changes in QTc, PQ and PFVd are common in young children undergoing surgery for ASD repair. Post-op QTc significantly correlates with bypass time, suggesting prolonged cardiopulmonary bypass may impair ventricular repolarization. Post-op QTc significantly correlates with PFVd changes, suggesting increased coronary flow may also impair ventricular repolarization. The clinical significance and reversibility of these alternations require further investigations.

## Background

Cardiopulmonary bypass remains the main approach to surgical repair of most congenital heart diseases. Little, however, is known about potential hemodynamic and electrophysiological consequences of this procedure, especially in young children. Myocardial function and output are usually impaired in congenital heart disease, but may become more so after surgery. Coronary flow increases after cardiopulmonary bypass surgery [1,2]. In adults, impaired myocardial repolarization and increased risk of arrhythmia are well known consequences of open heart surgery [3]. Early postoperative arrhythmia occurs in about 14% children [4].

QTd (the difference between maximal and minimal QT intervals on a standard ECG) is a risk factor for post-operative ventricular arrhythmia in patients with Tetralogy of Fallot [5,6]. QTc and QTd increase in left ventricular diastolic dysfunction [7]. Prolonged QRS duration, on the other hand, may result from right or left ventricular dilatation, predicting malignant ventricular arrhythmias [8]. PQ interval increases in diastolic dysfunction, independent of the severity or cause. Moreover, P wave dispersion correlates with left ventricular ejection fraction and is significantly higher in dilated cardiomyopathy and mitral stenosis [9]. Of note, the pattern of ventricular repolarization in male and female infants and children is the same [10,11].

We recently reported that cardiopulmonary bypass surgery in children leads to increased coronary flow in the first week of surgery [1]. Profound adverse effects (e.g., atrial fibrillation and ventricular arrhythmia) on myocardial repolarization in the same period are also reported in adults [12]. The primary aim of this study was to compare myocardial repolarization (QTc, QTd, and PQ intervals) and coronary flow (PFVd) measurements before and within the first week after cardiopulmonary bypass surgery for ASD repair in children. The secondary aim was to correlate the ECG and PFVd changes with bypass and aortic cross-clamp times.

## Methods

### Study population

Twelve children who had open heart surgery under cardiopulmonary bypass for ASD repair were included in this study. The mean age was 3.5 ± 1.9 years (range, 1–7). The study was approved by the ethics committee for human research at the Lund University. Written consent was obtained from the guardians/parents of patients enrolled in the study. The study protocol conformed to the principles outlined in the declaration of Helsinki [13]. The exclusion criteria were clinical signs of an infectious illness, C-reactive protein >0.8 mg/L before surgery, heart failure, and preoperative therapy with vasoactive drugs.

The cardio pulmonary bypass surgeries were performed under a body temperature of 28-32°C; cold (+4°C) hyperkalemic blood cardioplegic solution was used. All patients had the same protocol for cardioplegia and anterograde perfusion of the coronary arteries with retrograde filling through the coronary sinus every 20 min. The post-operative intensive care stay was uneventful with no observed arrhythmias.

Patients were not taking beta-blockers, angiotensin 1-converting enzyme inhibitors, angiotensin 1-converting enzyme receptor blockers, lanoxine, phenylepherine or other vasoactive drugs (including nitrate or dopamine). Patients received intravenous ketobemidone hydrochloride for pain during the first 2–3 days. Oral oxicodon, with a half-life of 4.5 hours, was then given on postoperative days 3 and 4. These medications were stopped at least one day before postoperative evaluations (echocardiography and ECG) on day 5 or 6, which were performed when patients were ready for discharge.

### ECG measurements

Surface (12-lead) electrocardiograms (ECG Machine-GE Marquette, 12 lead ecg system) were done one day before and 5 or 6 days after surgery. PQ interval and indexes of myocardial repolarization (QTc and QTd) were measured by one investigator. QT was measured manually in leads II, V4, and V5. QTc was calculated using the Bazett’s formula [14]. Three consecutive ECG cycles (whenever possible) in each of the three leads were measured and the mean interval was used. The precedent RR interval to the measured QT was used to calculate the heart rate for QTc interval in milliseconds (ms). QTd was set as the difference between the maximum and minimum QTc, using lead V1 [5].

### Transthoracic Doppler echocardiography

Echocardiographic examinations were made with Sequoia™ C512 (Acuson Mountain View, CA, USA) machine with pulsed wave Doppler frequency of 7/10 MHz. Standard M- and B-modes and 2D transthoracic Doppler Echocardiography (TTDE) for cardiac output, left ventricular function and left anterior descending (LAD) coronary artery flow measurements were done one day before and 5 or 6 days after surgery. The diameter of the aortic ring was measured in a long axis view by M-mode in accordance to the recommendations of the American Society of Echocardiography [15]. Left ventricular fractional shortening was computed according to the standard formula [16]. The flow velocity measurements across aortic valve were averaged over three consequent cardiac cycles. Adjustments in the ultrasound machine for measuring diastolic peak flow velocity (PFVd) in the LAD coronary artery were as previously described [1].

### Statistics

The data were expressed as mean and standard deviation (±SD). Analyses (standard crosstabs, Pearson correlation coefficients, and linear regression) were performed using SPSS version 20.0 (SPSS Incorporated, Chicago, Illinois). *P*-values <0.05 were considered significant.

Normality of PFVd, QTc, QTd and PQ changes was confirmed by the Shapiro-Wilk test. Thus, the paired t-test was used to compare pre- and post-operative changes in the measured parameters.

Adequacy of the sample size (n = 12) for detecting true changes, if any, between the pre- and post-operative values of PFVd, QTc, QTd and PQ by paired t-test was assessed. For this purpose, true standard deviations of differences were assumed to be close to the observed standard deviations. Smallest mean differences (pre- vs. post-operative) for detecting with a power of 90% and 5% significance were computed. The smallest mean difference for PFVd was 13.28 cm.s^-1^ (assumed standard deviation = 13.0 cm.s^-1^), for QTc was 20.59 ms (assumed standard deviation = 20.0 ms), QTd 15.44 ms (assumed standard deviation = 15.0 ms), and for PQ was 25.74 ms (assumed standard deviation = 25.0 ms).

## Results

All children, except patients 11 and 12, had normal myocardial repolarization (QTc and QTd intervals) before and after surgery. Patient **11** had the longest aortic cross-clamp duration (67 min), bypass time (106 min), pre-operative PQ interval (200 ms) and pre-operative QTc (444 ms); PQ and QTc decreased after surgery by 6% and 3%, respectively (Table 1).

**Table 1:** **Study patients, cardiopulmonary bypass (perfusion) time, aortic cross-clamp time, coronary flow velocity (PFVd, in cm.s**^**-1**^**), and pre- and post-operative (6 ± 1 days) QTc, QTd and PQ measurements**

**Patient**	**Age (mo) gender diagnosis**	**Bypass time (min)**	**Aortic cross-clamp time (min)**	**Pre-op LAD PFVd**	**Post-op LAD PFVd**	**Δ**	**Pre-op QTc (ms)**	**Post-op QTc (ms)**	**Δ**	**Pre-op QTd (ms)**	**Post-op QTd (ms)**	**Δ**	**Pre-op PQ (ms)**	**Post-op PQ (ms)**	**Δ**
1	72, F ASD-SV	82	39	50	65	+15 (30%)	412	409	-3 (<1%)	40	30	-10 (25%)	138	132	-6 (4%)
2	36, F ASD-2	58	14	43	67	+24 (56%)	394	393	-1 (<1%)	40	60	+20 (50%)	130	136	+6 (5%)
**3**	48, F ASD-2	168	26	40	90	+50 (125%)	415	451	+36 (9%)	30	50	+20 (67%)	150	194	+44 (30%)
4	60, F ASD-2	46	21	28	70	+42 (150%)	427	419	-8 (2%)	60	40	-20 (33%)	136	118	-18 (13%)
5	24, M ASD-2	64	31	36	57	+21 (58%)	374	392	+18 (5%)	40	40	0	134	144	+10 (7%)
6	15, F ASD-2	73	53	49	70	+21 (43%)	411	384	-27 (7%)	40	30	-10 (25%)	164	130	-34 (21%)
7	32, M ASD-2	108	60	53	93	+40 (75%)	413	418	+5 (1%)	60	60	0	130	92	-38 (29%)
8	12, M ASD-2	na	na	56	62	+6 (11%)	413	369	-44 (11%)	40	30	-10 (25)	136	100	-36 (26%)
9	84, M ASD-2	52	24	50	66	+16 (32%)	398	390	-8 (2%)	60	40	-20 (33%)	138	124	-14 (10%)
10	24, M ASD-1	53	26	42	52	+10 (24%)	375	401	+26 (7%)	30	40	+10 (33%)	134	134	0
**11**	48, M ASD-1	106	67	48	73	+25 (52%)	444	432	-12 (3%)	40	60	+20 (50%)	200	189	-11 (6%)
12	48, F ASD-2	56	25	55	85	+30 (54%)	421	448	+27 (6%)	40	20	-20 (50%)	126	150	+24 (10%)

*ASD* Atrial septal defect, *LAD* Left anterior descending artery, *PFVd* Peak flow velocity in diastole, *SV* Sinus venosus, *1* Primum, *2* Secondum, *QTC* QT Corrected, *QTd* QT Dispersion, *na* Not available.

**Figure 1:**
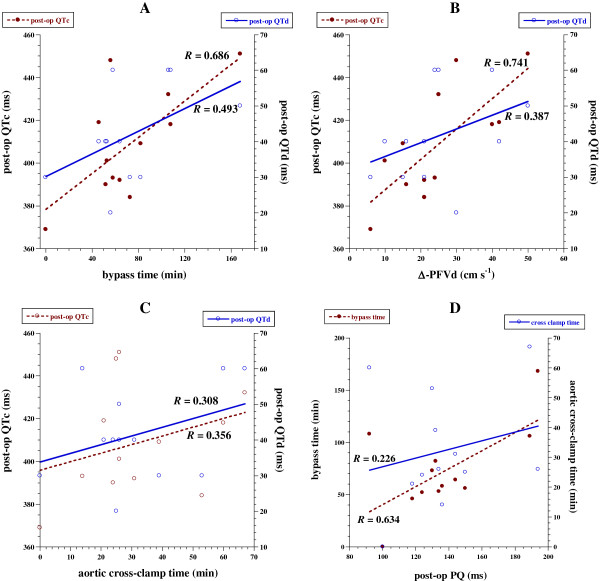
**A-D.** Correlations between post-operative ECG parameters vs. cardiopulmonary bypass time, Δ-LAD PFV and aorta cross clamp time. **A**; Correlation between post-op QTc and QTd and bypass time. **B**; Correlation between post-op QTc and QTd and Δ-LAD PFV. **C**; Correlation between post-op QTc and QTd and aortic cross-clamp time. **D**; Correlation between post op PQ duration and bypass time and aortic cross-clamp time.

Patient **12** had long post-operative QTc (448 ms) with relatively short aortic cross-clamp time (25 min). This patient had post-operative increase in PQ interval (by 10%), but still within the normal limits. These parameters increased 6-12% compared with the pre-op values. After surgery, QTc increased 1-9% in 5 of 12 (42%) patients, decreased 2-11% in 5 of 12 (42%) patients and did not change in 2 of 12 (16%) patients (Table 1). Post-op QTc positively correlated with bypass time (*R*=0.686, *p*=0.014) and changes in PFVd (*R*=0.741, *p=*0.006), Figure 1A-C.

After surgery, QTd increased 33-67% in 4 (33%) patients, decreased 25-50% in 6 (50%) patients and did not change in 2 (16%) patients. The correlations between post-op QTd and PFVd or aortic cross-clamp time were insignificant (Figure 1A-C).

After surgery, PQ interval increased 5-30% in 4 (33%) patients, decreased 4-29% in 6 (50%) patients and did not change in 1 (8%) patient. Post-op PQ positively correlated with bypass time (*R*=0.634, *p*=0.027), Figure 1D. The correlations between post-op PQ and PFVd or aortic cross-clamp time were insignificant (Figure 1D).

LAD coronary artery PFVd significantly increased after surgery (*p*<0.001). Otherwise, the paired t-tests for QTc, QTd and PQ were insignificant.

Hemodynamic and echocardiography data before and after surgery showed no significant changes, except for the systolic blood pressure (*p*=0.04, Table 2). Cardiac output and its related velocity time integral in the aorta were insignificantly decreased (*p* = 0.302 and 0.770, respectively) after surgery (Table 2). Left ventricular fraction shortening was the same before and after surgery. There were no significant correlations between postoperative cardiac function (including cardiac output) and myocardial repolarization parameters.

**Table 2:** Hemodynamics, ECG and echocardiography data before and after (6 ± 1 days) cardiopulmonary bypass surgery for ASD patients (n = 12)

	**Pre-surgery**	**Post-surgery**
HR, bpm	96 (13)	84 (22)
SBP, mmHg	104 (10)	101 (10)
DBP, mmHg	52 (8)	51 (7)
RPP, mmHg/bpm	9863 (1286)	8532 (2408)
***ECG***		
QT, ms	338 (35)	345 (45)
QTc, ms	408 (20)	408 (26)
QT dispersion, ms	43 (11)	42 (13)
PQ, ms	143 (21)	156 (89)
P duration, ms	93 (12)	112 (110)
***Echocardiography***		
FS, %	39 (7)	39 (4)
Aorta VTI, cm	19 (4)	18 (5)
CO, mL·min^–1^·kg^–1^	450 (136)	410 (150)
LVM, g	12 (3)	13 (4)
Aortic cross clamp time, min	-	35 (17)
CPB, min	-	78 (36)

Values are mean (±SD).

*Aorta VTI* Aorta velocity time integral, *BP* Blood pressure (in mm Hg), *HR* Heart rate (in beats per min), *RPP* Rate pressure product (beats × mmHg ÷ min), *FS* Fractional shortening, *LVM* Left ventricular mass, *CO* Cardiac output, *CPB* Cardiopulmonary bypass, *VTId+s* Velocity time integral in diastole and systole, *BF* Blood flow.

## Discussion

The study describes early changes in ventricular repolarization (QTc, QTd), PQ interval and coronary flow (PFVd) shortly after cardiopulmonary bypass for ASD repair in children. This surgical procedure was associated with significant changes in these parameters in most patients. Some changes were partially related to the bypass and aortic cross-clamp times (Figure 1). Thus, prolonged intra-operative procedure may have an adverse impact on myocardial repolarization, imposing some risk of arrhythmia after surgery. This possibility needs further investigation, especially with extended time following surgery.

The cross clamp duration shown here is comparable with that reported for ASD repair (35 ± 17 *vs*. 24 ± 16 min) [17]. Three of our 12 patients had associated congenital heart defects, such as partial anomalous pulmonary venous return. For these children, the cardiopulmonary bypass time was >100 min. Otherwise, the bypass time for the remaining 8 children was 61 ± 12 min, which is comparable with published report (42 ± 16 min) [17].

ASD causes right atrial dilatation and right ventricular volume overload. These alterations along with incision made through the right atrium and bypass time during surgery likely impact the myocardial repolarization. The overall effects are variable and their clinical significance remains uncertain. Further studies are needed to address reversibility and significance of these changes.

Impaired myocardial repolarization (and its associated risk of arrhythmia) in the immediate post-operative period perhaps could be deduced from the significant correlations between duration of bypass and post-op QTc or PQ intervals (Figure 1). A recent study of various congenital heart diseases showed persistently prolonged QTc >3 days after surgery in about one-third of the patients [18]. A transient prolongation of QTc is also reported in adults during myocardial infarction and balloon coronary angioplasty [19,20].

The post-operative increase of coronary artery flow could have a mechanistic link to ventricular repolarization, accounting (at least partially) for the significant correlation between PFVd changes and post-op QTc (Figure 1B). This finding is in line with the animal study by Zhang et al., where increased coronary flow was associated with prolonged duration of transmural ventricular repolarization [21]. The underlying mechanism of flow-induced lengthening of ventricular repolarization is unclear, but synthesis and release of nitric oxide from the coronary endothelium may play a role [22]. The right ventricular volume and pressure are increased in ASD, raising the demand for oxygen. The coronary flow, thus, is expected to increase in these patients. These findings become more prominent after cardiopulmonary bypass surgery [23]. Patients with limited coronary flow reserve (e.g., prolonged aortic cross-clamping duration and enhanced microcirculatory changes), however, may fail to execute adequate oxygen supply to the myocardium. Such patients may have complicated post-op course, particularly with sepsis. Therefore, assessing coronary flow is important in critically-ill children.

## Conclusions

Changes in myocardial repolarization (QTc), PQ interval and coronary perfusion (PFVd) are common in young children undergoing cardiopulmonary bypass surgery for ASD repair. Post-op QTc significantly correlates with bypass time, suggesting prolonged cardiopulmonary bypass may impair ventricular repolarization. Post-op QTc also significantly correlates with PFVd changes, suggesting the post-op increase in coronary flow may impair ventricular repolarization. The clinical significance and reversibility of these alternations require further investigations.

### Limitations of the study

The study was conducted in a small cohort. To avoid stress on child and parents, the coronary flow reserve was not done.

## Abbreviations

ASD: Atrial septal defect; QTc: Corrected QT interval; QTd: QT dispersion; LAD: Left anterior descending; PFVd: Peak flow velocity in diastole; ECG: Electrocardiogram; TTDE: Transthoracic doppler echocardiography; SD: Standard deviation; SV: Sinus venosus; VTI: Velocity time integral; VTId+s: Velocity time integral in diastole and systole; BP: Blood pressure; HR: Heart rate; RPP: Rate pressure product; FS: Fractional shortening; LVM: Left ventricular mass; CO: Cardiac output; CPB: Cardiopulmonary bypass; VTId+s: Velocity time integral in diastole and systole; BF: Blood flow; TTDE: Transthoracic doppler echocardiography.

## Competing interests

There are no potential, perceived, or real conflicts of interest.

## Authors’ contributions

EHA, PL and EP designed the study and get the ethical approval. EHA and A-KS analyzed the data and prepared the first draft of the manuscript. PL and EP revised and edited the manuscript. TZ performed the statistical analysis. All authors have read and approved the final version of the manuscript.

## Pre-publication history

The pre-publication history for this paper can be accessed here:

http://www.biomedcentral.com/1471-2261/13/67/prepub
